# Cisplatin in Ovarian Cancer Treatment—Known Limitations in Therapy Force New Solutions

**DOI:** 10.3390/ijms24087585

**Published:** 2023-04-20

**Authors:** Aleksandra Zoń, Ilona Bednarek

**Affiliations:** Department of Biotechnology and Genetic Engineering, Faculty of Pharmaceutical Sciences in Sosnowiec, Medical University of Silesia, Jedności 8, 41-200 Sosnowiec, Poland

**Keywords:** cisplatin, ovarian cancer, drug resistance, combination therapy, cisplatin analogs

## Abstract

Cisplatin is one of the most commonly used anticancer drugs worldwide. It is mainly used in the treatment of ovarian cancer, but also used in testicular, bladder and lung cancers. The significant advantage of this drug is the multidirectional mechanism of its anticancer action, with the most important direction being damaging the DNA of cancer cells. Unfortunately, cisplatin displays a number of serious disadvantages, including toxicity to the most important organs, such as kidneys, heart, liver and inner ear. Moreover, a significant problem among patients with ovarian cancer, treated with cisplatin, is the development of numerous resistance mechanisms during therapy, including changes in the processes of cellular drug import and export, changes in the DNA damage repair mechanisms, as well as numerous changes in the processes of apoptosis and autophagy. Due to all of the mentioned problems, strategies to increase the effectiveness of cisplatin in the treatment of ovarian cancer are intensively sought. The most important strategy includes the development of less toxic cisplatin analogs. Another important direction is combination therapy, involving the simultaneous use of cisplatin with different anticancer drugs, substances derived from plants, temperature or radiotherapy. Many years of observations accompanying the presence of cisplatin in the therapy made it possible to provide a series of verifiable, statistically significant data, but also to show how, over time, with the new information and scientific discoveries, it is possible to describe and understand the therapeutic problems observed in practice, such as the acquisition of drug resistance by tumor cells or induction of changes in the tumor microenvironment. According to the authors, confronting what we knew so far with what new trends offer has a profound meaning. This paper presents information on the history of cisplatin and describes the molecular mechanisms of its action and the development of resistance by cancer cells. In addition, our goal was to highlight a number of therapeutic strategies to increase the effectiveness of cisplatin in the treatment of ovarian cancer, as well as to identify methods to eliminate problems associated with the use of cisplatin.

## 1. Introduction

Ovarian cancer is the third most common female cancer in the world. Data published in 2020 indicate that the disease has been diagnosed in nearly 314,000 women worldwide [1]. It is estimated that by 2040, the number of women suffering from ovarian cancer will increase to over 445,000 [2]. The 5-year survival rate of patients who have been diagnosed with this disease is 45.6%; however, it increases to about 70% if the disease is detected at an early stage. Unfortunately, data show that only 20% of cases are diagnosed at an early stage of cancer development [3,4]. At present, the basic treatment of ovarian cancer is the surgical removal of the tumor mass, combined with radio- or chemotherapy. In addition, targeted therapy, hormonal therapy and immunotherapy are also used in the treatment [3].

Despite the emergence of new, more advanced forms of therapy for this cancer, the most common form of treatment is still pharmacotherapy which is based on compounds from the taxane family, paclitaxel or docetaxel, and on platinum-containing compounds, such as cisplatin, which is the subject of this paper [3,5].

Discovered in 1845 by Michele Peyrone, cisplatin was introduced for the treatment of cancer in 1978, as the first-ever anticancer drug containing a metal ion. Currently, cisplatin is used in the therapy of about 50% of all cancer patients and in the treatment of, among others, ovarian, testicular, lung, bladder, breast and gastric cancers [6,7,8]. Such a wide spectrum of use of this drug results from the multidirectional mechanism of its action, including damaging genetic material, increasing the production of superoxides in cells and damaging mitochondrial DNA [6,9]. Unfortunately, cisplatin is not without its drawbacks. The most important ones are side effects caused by this drug, such as nephrotoxicity, ototoxicity and gastrointestinal toxicity. Another significant disadvantage of cisplatin is the development of resistance to its action during the therapy by cancer cells [10,11,12,13]. It has been proven that cancer cells develop a number of mechanisms that protect them from the cytotoxic effect of cisplatin, such as changes in DNA damage repair mechanisms or changes in the function of membrane transporters [14,15,16]. All of the aforementioned problems have resulted in intense searches to increase the effectiveness of cisplatin, reduce its toxicity and overcome the mechanisms of cell resistance. Recent studies indicate that such methods may include combined therapy of cisplatin and hyperthermia or radiotherapy, as well as a combination of cisplatin with compounds of plant origin, and other drugs [17,18,19,20].

The following article presents the history of cisplatin introduction into treatment and the mechanisms of its anticancer action. Problems associated with the use of cisplatin, such as the toxicity of its action or the development of resistance mechanisms by cancer cells, have also been described. In addition, this paper discusses the topic of the latest therapeutic strategies that allow for increasing the efficiency and effectiveness of the drug’s action.

## 2. The History of Cisplatin

Cisplatin is a complex compound with a planar structure. It contains two chlorine atoms and two ammonia molecules, linked in a cis configuration to the platinum atom in the second oxidation state, by a coordination bond [21].

The discovery of the anticancer activity of cisplatin was accidental. In 1965, the American biophysicist Barnett Rosenberg conducted research on the effect of different frequencies of alternating current on the ability of *Escherichia coli* cells to divide. During his research, the scientist found out that the use of electric current leads to the formation of a complex compound on the surface of the platinum electrodes placed in the ammonium buffer, which had the ability to inhibit cell division. That compound was later known as cisplatin [22,23,24,25]. The later continued research indicated the ability of cisplatin to inhibit the division of cancer cells—sarcoma cells (cell line S180) and leukemia cells (cell line L1210), as well as the ability to reduce tumor weight in rats with sarcoma [22,24,25]. Because of safety concerns, cisplatin was not introduced into human therapy until 1978, when the pharmaceutical company Bristol-Myers conducted a number of additional studies on the safety of the drug, which provided results that allowed cisplatin to be registered by the Food and Drug Administration as a drug that can be used in cancer therapy [22].

**Figure 1:**
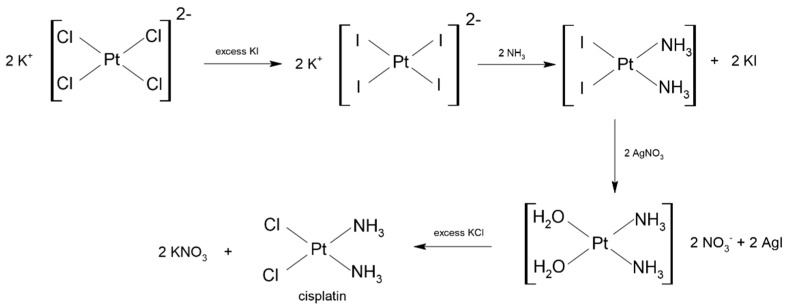
Scheme of cisplatin synthesis by Dhara’s method.

Nowadays, one of the most frequently used methods of cisplatin synthesis is Dhara’s method developed in 1970. It is a multi-stage method, the first step of which is the conversion of K_2_[PtCl_4_] to K_2_[PtI_4_] by treating the salt with an excess of KI. Then, to the resulting dark brown solution of K_2_[PtI_4_], ammonia water is added, which results in the precipitation of a yellow cis-[Pt(NH_3_)_2_I_2_] precipitate, which is then collected and dried. The next stage of the synthesis is removing the iodide ligands from the compound by adding an aqueous solution of AgNO_3_, which results in the formation of soluble [Pt(NH_3_)_2_(H_2_O)_2_]^2+^ and insoluble AgI, that is filtered out. In the final step of the synthesis, the filtrate containing [Pt(NH_3_)_2_(H_2_O)_2_]^2+^ is treated with excess KCl, to give isomeric pure cisplatin in a form of a yellow solid [26]. The scheme of the described reaction is shown in Figure 1.

## 3. Cisplatin—Mechanisms of Its Cytotoxic Action

Cisplatin is administrated to patients intravenously, as a solution in saline. While circulating in the bloodstream, the drug can be easily bonded by amino acids and proteins that are present in the blood (for example, cysteine or albumin), which results in the inactivation of 65–95% of the cisplatin administered to the patient’s body. Cisplatin that has not been deactivated is imported into cells by the membrane transport proteins CTR1 and CTR2 and by passive diffusion. Once inside the cells, cisplatin is activated by exchanging one or two chloride ligands for water [27,28]. The hydrated and activated cisplatin is a compound with a multidirectional cytotoxic activity, including inducing oxidative stress in cells, as well as damaging cellular and mitochondrial DNA in cancer cells [6,29].

### 3.1. Damaging the Genomic DNA

The main target of cisplatin’s cytotoxic effect is genomic DNA. After entering the cell, cisplatin binds to nitrogenous bases in DNA—most efficiently to guanine and adenine at the N7 position of their imidazole ring [6,30,31,32]. Most frequently, it binds to two guanines on the same DNA strand (65% of cases), or to guanine and adenine on the same strand (25% of cases), which results in the formation of intrastrand adducts. Much less often, only in 5% of cases, the drug binds to two guanines located on the opposite strands, which leads to the formation of interstrand crosslinks [30,31,32]. The formation of both intrastrand adducts and interstrand crosslinks leads to the damage of the genetic material. DNA damage caused by cisplatin activates DNA repair mechanisms, mainly the nucleotide excision repair (NER) mechanism, as well as the mismatch repair (MMR) mechanism. When these mechanisms prove ineffective, activation of apoptosis pathways occurs in the cell [6,33]. Activation of these pathways is possible, for example, by inhibition of the cell cycle in the G1, S or G2 phase, which leads to the lack of DNA synthesis in the cell, and, as a result, to its death [3,33].

### 3.2. Damaging the Mitochondrial DNA

Another target of cisplatin action is the mitochondrial DNA. Same as with the cellular DNA, the drug binds to the mtDNA, leading to the formation of adducts and crosslinks and, consequently, to its damage [34]. Damage to the mitochondrial DNA results in a change in the permeability of the mitochondrial membranes, which leads to the release of cytochrome C and procaspase 9 from the organelle. The components released from the mitochondria bind with APAF-1 and ATP to form an apoptosome, which then activates caspase 9. Activated caspase 9 is interacting with subsequent caspases, leading to the activation of caspases 3, 6 and 7, which induce apoptosis of the cell [6,35].

### 3.3. Induction of Oxidative Stress in Cells

Another mechanism of cisplatin’s cytotoxic action is the induction of oxidative stress in cells by increasing the production level of reactive oxygen species, such as hydroxyl radicals and superoxides.

It is assumed that there are three main intracellular compartments, where cisplatin induces the formation of ROS: cell membrane, cytoplasm and cell organelles [36]. In the cell membrane, a large amount of reactive oxygen species causes the activation of acid sphingomyelinase, which, by hydrolyzing sphingolipids to ceramides, leads to the clustering of FAS receptors in the plasma membrane, leading to cell death [36,37]. The organelles that are the most exposed to ROS are mitochondria. Excess ROS, interacting with pro-apoptotic BAX protein, are damaging and changing the potential of the mitochondrial membrane, thus leading to the activation of apoptosis by the previously described intrinsic pathway [36,38].

## 4. Disadvantages and Limitations of Cisplatin

Despite the wide range of anticancer activity of cisplatin, which allows to use it in the treatment of many types of cancer, its actual use in the treatment of patients is limited due to a number of disadvantages. The most important of them include causing toxic side effects in the patient’s normal tissues, as well as the acquisition of cisplatin resistance by cancer cells during treatment, which significantly reduces the effectiveness of its action [39].

### 4.1. Toxic Side Effects

The main reasons for limiting the use of cisplatin in cancer patients are its dangerous side effects, which include nephrotoxicity (observed in 28–36% of patients), ototoxicity (observed in 10–90% of patients), neurotoxicity (observed in 30% of patients), gastrointestinal toxicity (observed in 70–80% of patients), as well as hepatotoxicity, cardiotoxicity and hematological toxicity that are observed much less often [11,12,13,39,40,41,42]. The side effects significantly reduce the quality of patients’ life and force a reduction in the dose of the drug or even its complete discontinuation, thus reducing the effectiveness of anticancer therapy [39,43].

### 4.2. Acquisition of Resistance to Cisplatin by Cancer Cells

**Figure 2:**
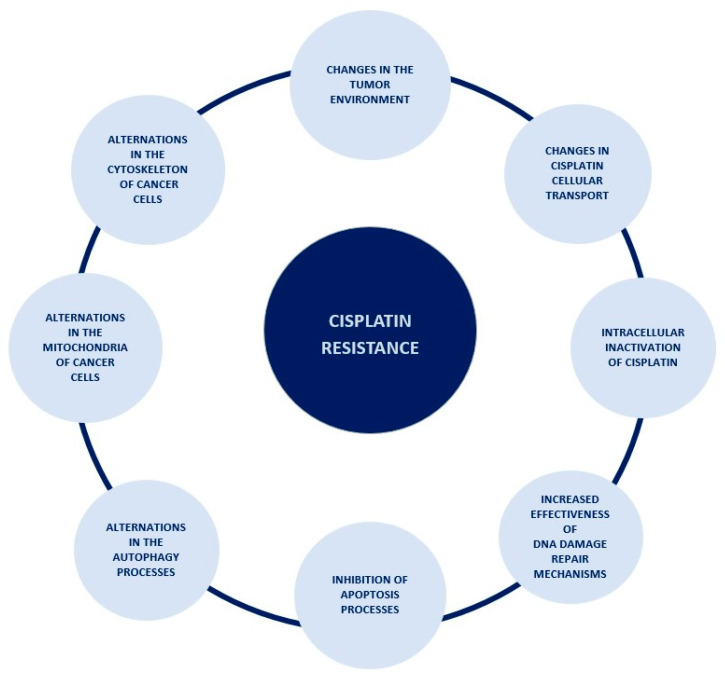
The spectrum of occurrences lying behind the mechanism of cisplatin resistance.

Resistance to a cytotoxic drug is defined as the lack of activation of apoptosis in cancer cells, despite the use of a drug at doses that usually cause apoptosis of these cells [38]. There are two types of resistance: innate, which occurs without prior exposure to the drug, and acquired, which is the result of the exposure to the drug [43]. The process of acquiring resistance to cisplatin is a very complex and multifactorial process, and this article describes only a part of the mechanisms taking place in ovarian cancer cells leading to its occurrence. All mechanisms discussed in this paper were summarized in Figure 2.

#### 4.2.1. Changes in Cisplatin Cellular Transport

One of the mechanisms developed by cisplatin-resistant cells is the reduction in the intensity of cellular uptake of the drug.

Cisplatin is transported into the cells mainly in the process of passive diffusion, as well as by the membrane transporters—CTR1 and CTR2. The aforementioned CTR1 and CTR2 transporters are proteins belonging to the superfamily of membrane-spanning protein transporters, encoded respectively by the SLC31A1 and SLC7A2 genes [16,44]. These proteins are responsible for the process of transporting copper ions into the cells and thus for maintaining copper ion homeostasis in the cells. In addition, it has been shown that they are also involved in the import of cisplatin into cells. The transporter that is directly responsible for the transport of metal ions is CTR1—it binds and then imports copper and platinum ions into the cells, by means of the metal-binding ectodomain [44,45,46]. The CTR2 protein acts as a regulator for the CTR1, which, by cleaving the aforementioned ectodomain, reduces its affinity for copper and platinum ions, thus changing the intensity of intracellular transport of both copper ions and cisplatin [44,47].

Numerous studies have shown that in cisplatin-resistant cells, there are changes in the activity of mentioned transporters, resulting in a reduced influx of the drug into the cells [48]. In cisplatin-resistant cells, there is a significant decrease in the level of CTR1 protein and an increase in the level of CTR2 protein, which results in a decrease in cisplatin uptake by these cells and a significant decrease in its effectiveness [48]. It has also been established that in patients with ovarian cancer, high levels of mRNA encoding the CTR1 protein correlate with longer disease-free survival, while low levels of the CTR2 protein correlate with better outcomes of cisplatin therapy [48]. In addition, it is suggested that the determination of the CTR1/CTR2 protein ratio may be a useful biomarker to determine the cisplatin sensitivity of a tumor [49].

Another mechanism developed by cisplatin-resistant cells is the increased export of cisplatin. Cisplatin is transported out of the cells by ATP7A and ATP7B transporters, which are classified into the group of heavy metals transporting P-type ATPases [50]. The most important function of these transporters is the export of copper ions out of the cells, which is possible due to the presence of metal-binding domains in their structure that enable the attachment and transport of metal ions outside the cells. In addition, these transporters have the ability to transport platinum ions, which makes them the most important mechanism for the export of cisplatin from the cells [50].

In ovarian cancer cells, an increased expression of both of these transporters is observed, which results in an increase in the intensity of drug transport from the cells, a decrease in the level of accumulated drug in cancer cells and, as a result, an increase in resistance to treatment [51,52]. Due to the strong correlation between the activity of the ATP7B transporter and resistance to cisplatin, this protein can be used as a biomarker of tumor resistance to this drug [53,54].

#### 4.2.2. Intracellular Inactivation of Cisplatin

Another mechanism of resistance to cisplatin developed by cancer cells is intracellular inactivation of the drug, which leads to a reduction in the amount of drug that is capable of binding to DNA. After activation inside cells, cisplatin can bind not only to DNA, but also to other nucleophilic cellular components, e.g., glutathione or metallothioneins, which causes its inactivation and loss of cytotoxic effect [14,55]. Numerous studies have shown that, in ovarian cancer cells, there is a significant increase in the level of glutathione and π-class glutathione transferase, which catalyzes the conjugation of cisplatin with glutathione [14,55,56]. The formation of cisplatin—glutathione conjugates causes the inactivation of the drug and increases its solubility, thus increasing its excretion from cells and reducing its cytotoxic effect [43].

In the same way as with glutathione, cisplatin can be inactivated by binding to metallothioneins. Overexpression of metallothionein I and II is very often observed in patients treated with cisplatin and patients with recurrent ovarian cancer, unresponsive to therapy with this drug [43,57].

#### 4.2.3. Increased Effectiveness of DNA Damage Repair Mechanisms

One of the most important mechanisms of resistance developed by cisplatin-resistant ovarian cancer cells is the increase in the effectiveness of DNA damage repair mechanisms. Damages of genetic material, caused by cisplatin, are mainly repaired by two mechanisms: nucleotide excision repair (NER) and mismatch repair (MMR) [34].

The most important mechanism involved in the repair of DNA damage is NER, responsible for the removal of cisplatin–DNA adducts. In the first stage of repair, XPC-HR23B proteins recognize DNA damage, then the DNA strands are separated by XPD and XPB helicases, and the DNA strand is cut on both sides of the adduct by XPF/ERCC1 and XPG endonucleases. Finally, about 30 nucleotides (including the adduct) are excised, and the resulting gap is filled by DNA polymerase ε [30]. It has been found that in the cisplatin-resistant cells, expression of the ERCC1 protein is upgraded, which results in an increased capacity of these cells to repair cisplatin-induced DNA damage [34,58].

Another mechanism involved in the repair of DNA damage is MMR, responsible for correcting errors in one of the DNA strands, such as mismatches, insertions or deletions of bases during replication. Additionally, this mechanism also recognizes changes caused by alkylating agents, including cisplatin [30]. Unfortunately, because this mechanism is only able to replace the base opposite of the DNA—cisplatin adduct, it cannot completely remove the damage. This leads to a restart of the whole repair process, which can eventually lead to a double-strand break in the DNA, activation of damage signaling proteins (including p53) and consequently to activation of the apoptosis process [59]. Cisplatin-resistant ovarian cancer cells have been observed to downregulate the MMR mechanism by mutations or by methylation of promoters of genes encoding proteins involved in this mechanism [30,59,60].

#### 4.2.4. Inhibition of Apoptosis Processes

One of the mechanisms of resistance to cisplatin developed by cancer cells is the inhibition of apoptosis processes induced by the drug. Studies show that cisplatin-resistant cells overexpress the antiapoptotic protein BCL-2, which results in a decrease in the BAX:BCL-2 ratio and inhibition of the apoptotic process activated by cisplatin [14,61]. In addition, cisplatin-resistant cells overexpress proteins from the IAP (inhibitors of apoptosis) family—mainly survivin and XIAP (X-linked inhibitor of apoptosis). These proteins inhibit the activation of caspases, primarily caspases 3, 8 and 9, preventing the activation of apoptosis by cisplatin [14,62].

#### 4.2.5. Changes in the Autophagy Processes

In recent years, scientists started to pay a lot of attention to the role of the autophagy process in the resistance of ovarian cancer cells to cisplatin [38].

Autophagy is a process that occurs in eukaryotic cells under conditions of nutrient deficiency or stress. It is a process involving the removal of misfolded proteins, nonfunctioning organelles or cellular debris from cells. These components are engulfed by autophagosomes—vesicular structures made of a double lipid membrane and then transported to lysosomes, where they are degraded. The compounds obtained in this way can be reused by the cell [38]. Changes in the apoptosis process observed in cisplatin-resistant ovarian cancer cells are one of the factors that increase the survival of these cells despite the use of therapy. It has been established that in the described cells, the activation of the autophagy process is facilitated as a result of cellular stress, which in a way “replaces” the activation of cell apoptosis processes, contributing to the reduction in the effects of cisplatin and thus to the survival of cancer cells [38,63].

#### 4.2.6. Changes in the Mitochondria of Cancer Cells

As emphasized by recent studies, changes in the mitochondria of ovarian cancer cells, for example, changes in the number of mitochondrial DNA copies, have a large impact on the resistance of these cells to cisplatin [64]. It has been proven that in cisplatin-resistant cells, there is a significant increase in the number of mtDNA copies, which probably leads to an increase in the level of expression of antioxidant enzymes genes, allowing for an effective fight against ROS, generated as a result of cisplatin action [65].

In addition, in cisplatin-resistant ovarian cancer cells, changes in the dynamics of mitochondria have been observed. The mentioned cells overexpressed the OPA-1 protein, which mediates the process of mitochondrial fusion—the joining of two mitochondria to form one. The increase in the expression of the OPA-1 protein leads to an increase in the intensity of the mitochondrial fusion process compared to cisplatin-sensitive cells [66]. It is suggested that the process of mitochondrial fusion allows for maintaining their proper functioning under conditions of oxidative stress and, consequently, allows the survival of cells exposed to cisplatin [64,66,67].

At the same time, in ovarian cancer cells, there is a decrease in the expression of the DRP1 protein, which is involved in mitochondrial fission—the process of division of one mitochondrion into two, independently functioning mitochondria [68]. In this process, cytochrome c is released from the mitochondria, which, in subsequent stages, leads to apoptosis. Inhibition of mitochondrial fragmentation allows the survival of cisplatin-resistant cells [64,68]. 

#### 4.2.7. Changes in the Cytoskeleton of Cancer Cells

Another relatively recently discovered mechanism developed by cisplatin-resistant cells is the changes in the dynamics of their actin cytoskeleton. The actin cytoskeleton is a cellular structure involved in processes, such as cellular movement, intracellular transport and apoptosis. It is made of actin filaments formed as a result of twisting two G-actin strands, which undergo reversible polymerization and depolymerization during their cellular functions. The key regulators of the organization and dynamics of the actin skeleton are the RHO family proteins: RHOA, RHOB and RHOC [69,70].

As it turns out, cisplatin-resistant ovarian cancer cells show significant differences in the dynamics of the actin cytoskeleton compared to sensitive cells. The described cells are very often characterized by greater stiffness than normal cells, as well as by significant disorders within their cytoskeleton. The likely cause of these differences is the changes in the expression of RHO proteins—in cisplatin-resistant cells, there is an overexpression of RHOA and RHOC proteins and a simultaneous decrease in the expression of RHOB protein. Changes in the expression of mentioned proteins lead to depolymerization and impairment of the proper functioning of F-actin, which probably results in impaired function of membrane transporters, such as VSOR anion channels, leading to a decrease in the influx of cisplatin into the cells and thus to resistance to its action [69,71].

#### 4.2.8. Changes in the Tumor Microenvironment

In addition to the resistance mechanisms developed directly by ovarian cancer cells, scientists are also interested in changes in the microenvironment of cancer tumors, which may also contribute to the development of resistance to cisplatin [72].

The most important changes in the tumor microenvironment are the ones that limit the diffusion of cisplatin into the tumor, for example, the high density of tumor cells, or changes in the extracellular matrix, such as its increased rigidity [72,73,74].

An important role in the acquisition of resistance to cisplatin is played by noncancerous cells that are present in the tumor microenvironment, such as carcinoma-associated fibroblasts (CAFs) and tumor-associated macrophages (TAMs). It is established that these cells have the ability to inhibit the proapoptotic effect of cisplatin, by secreting numerous chemokines and growth factors that activate antiapoptotic pathways [75,76,77,78].

## 5. Strategies to Increase the Effectiveness of Cisplatin in the Treatment of Ovarian Cancer

Due to the growing problem of the development of resistance to cisplatin by ovarian cancer cells, methods of increasing the efficiency and effectiveness of the cytotoxic effect of cisplatin, as well as methods of inhibiting the mechanisms of resistance developed by cancer cells and restoring the sensitivity of these cells, are intensively sought. A particularly interesting strategy seems to be combination therapy—a therapy consisting of combining two or more agents with cytotoxic activity, for example, several drugs, or a drug with ionizing radiation or high temperature [5,19,20,79,80]. The examples of potential combination therapies with cisplatin were further discussed in the following subsections and summarized in Table 1.

Although platinum compounds are still the main therapy for ovarian cancers, it is absolutely important to note that histopathological heterogeneity guides the therapeutic approach in a very important way. For example, in the case of mucinous ovarian carcinoma or ovarian clear cell carcinoma, which usually demonstrates a poor response to cisplatin treatment, the molecular therapy targeting VEGF (e.g., Bevacizumab), EGFR (e.g., Gefitinib), SCR pathway (e.g., Dasatinib) or Her2 receptor (e.g., Trastuzumab) seems to be more suitable [81,82,83,84].

### 5.1. Designing of Cisplatin Analogs

Due to the number of side effects and the growing resistance phenomenon, there is an intensive sought for cisplatin analogs, which would be characterized by cytotoxic properties similar to cisplatin, but would be devoid of disadvantages limiting their widespread use. Currently, carboplatin and oxaliplatin are approved for treatment worldwide, and nedaplatin, heptaplatin and lobaplatin are in clinical trials [85]. A summary of approved and under-trial cisplatin analogs and their possible application is presented in Table 2.

**Figure 3:**
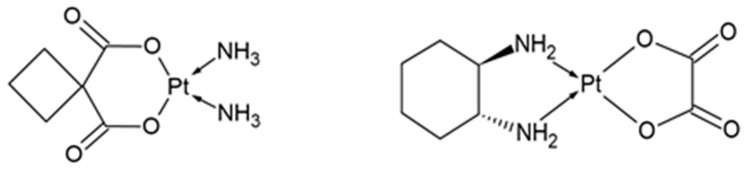
Chemical structure of carboplatin (**left**) and oxaliplatin (**right**).

Carboplatin is a second-generation platinum compound, introduced for the treatment of cancer in 1989 [85,86]. Its chemical structure is partially similar to the structure of cisplatin; however, in the place of chlorine atoms, there is an anion of cyclobutane-1,1-dicarboxylic acid (Figure 3). After entering the cell, the cyclobutane-1,1-dicarboxylic acid anion is replaced by a water molecule (just like the chlorine atoms in cisplatin), which activates the drug. The mechanism of action of carboplatin is analogous to the mechanism of action of cisplatin—the drug binds with nitrogenous bases in DNA, causing the formation of adducts, leading to DNA damage, which, in turn, results in the inhibition of replication and transcription processes, and finally cell apoptosis [85,87,88]. Both the cellular activation of carboplatin and its binding to DNA occur much slower than in the case of cisplatin, which, on the one hand, reduces the toxic side effects and ensures better tolerance of the drug by patients, but, on the other hand, reduces the effectiveness of the drug compared to cisplatin, resulting in the need of its higher doses [85,89].

Oxaliplatin is a third-generation platinum compound. Its structure is similar to that of carboplatin, but in place of the amine ligands, there is a 1,2-diaminocyclohexane group (Figure 3). The mechanism of action of oxaliplatin is the same as the mechanism of action of cisplatin and carboplatin and involves the formation of adducts with DNA [85,90]. This drug shows significantly lower systemic toxicity than cisplatin, but unfortunately also lower anticancer efficacy, which means that it is mainly used in combination with other chemotherapeutic drugs [91,92,93]. Currently, oxaliplatin is mainly used to treat colon, stomach and esophagus cancers, but studies show that it may be also effective in the treatment of ovarian cancer, particularly in patients who have relapsed or are hypersensitive to cisplatin or carboplatin [91].

The most important cisplatin analog, which is currently in clinical trials, is nedaplatin. Its structure is similar to cisplatin and carboplatin structures—it has two amine ligands and, additionally, a dianion of glycolic acid. Its mechanism of action is analogous to the previously discussed drugs [85]. The great advantage of the drug is the lack of nephrotoxicity, and its cytotoxic effect is similar to that of carboplatin [85]. Clinical studies have shown that nedaplatin is highly effective in the treatment of ovarian cancer recurrences previously treated with cisplatin [85,94].

### 5.2. Combination Therapy with Other Drugs

To overcome the problems associated with the development of cisplatin resistance and the relapses during the treatment of ovarian cancer, combination therapy, consisting of the use of two drugs with different mechanisms of action, is used.

An example of combination therapy that is most commonly used in the treatment of ovarian cancer is the simultaneous use of cisplatin with paclitaxel [95]. Paclitaxel is a compound belonging to the group of taxanes, it was first isolated from *Taxus brevifolia* in 1962, and introduced for the treatment of ovarian cancer, in 1992 [95]. Paclitaxel has the ability to bind to the microtubules of tumor cells, which results in the stabilization of microtubule bundles and inhibition of microtubule depolymerization. Improper functioning of microtubules prevents the formation of the metaphase plate, which leads to the inhibition of cell division at the metaphase/anaphase stage and thus to its apoptosis [96,97]. Since the 2000s, the combination of cisplatin with paclitaxel or carboplatin with paclitaxel has replaced platinum monotherapy as a first-line treatment for patients with advanced ovarian cancer. Studies show that the use of therapy that combines cisplatin with paclitaxel has a positive effect on overall survival and progression-free survival in patients with ovarian cancer [5,79].

A different example of a drug used in combination therapy of ovarian cancer is another substance from the taxane group—docetaxel. This drug is obtained from *Taxus baccata* as a product of a semisynthetic process. The mechanism of its anticancer activity is the same as that of paclitaxel, but its effect is 1.2–1.6 times stronger [98]. As in the case of paclitaxel, and also in the case of docetaxel, studies show that its use in combination with cisplatin significantly extends the overall survival of patients compared to monotherapy with cisplatin [98].

### 5.3. Combination Therapy with Substances Derived from Plants

Another strategy to increase the effectiveness of cisplatin action is a therapy using plant-derived compounds. Plant-derived compounds have a number of advantages that enable their use as supplementary substances in cancer therapy, including multiple mechanisms of anticancer activity, the ability to restore the sensitivity of cancer cells to cisplatin, causing minor side effects, as well as the ability to avoid most of the resistance mechanisms developed by cancer cells [17].

Compounds that can be used to increase the effectiveness of cisplatin belong mainly to the following groups: alkaloids, phenols and quinones.

An example of an alkaloid compound with anticancer activity is cardamonin—a substance obtained from plants belonging to the *Zingiberaceae* family, mainly *Alpinia conchigera* and *Alpinia katsumadai* [99]. It has been proven that cardamonin has the ability to inhibit the activity of mTOR kinase (the increased activity of which correlates with cisplatin resistance in ovarian cancer cells), which may lead to the restoration of sensitivity of cancer cells to cisplatin. In addition, it has been shown that the simultaneous treatment of ovarian cancer cells with cisplatin and cardamonin leads to inhibition of the cell cycle in the G2/M phase (by both substances) and thus potentiation of the antiproliferative effect of cisplatin. What is important is that the simultaneous use of cardamonin with cisplatin in low concentration gives the same effect as the use of high-concentration cisplatin in monotherapy, suggesting that the combined use of cisplatin and cardamonin may increase the efficiency of the cytotoxic effect of cisplatin and reduce the undesirable side effects of the drug, which occur when using it in high doses [99].

Another substance from the group of alkaloids that in combination with cisplatin can be used in the treatment of ovarian cancer is berberine—a substance that can be found, for example, in *Berberis vulgaris* L., *Mahonia aquifolium* or *Hydrastis canadensis* L. [100]. Studies show that berberine has the ability to increase the sensitivity of ovarian cancer cells to cisplatin by reducing the level of miR-93 in these cells. The lower level of miR-93 in cancer cells indirectly leads to the inhibition of the activity of the AKT pathway (participating in the development of cisplatin resistance) and, as a result, increases the sensitivity of ovarian cancer cells to cisplatin [100].

Another group of compounds with anticancer activity is quinones, represented by emodin. This compound, which can be found in *Rheum rhaponticum* L. or *Reynoutria japonica*, shows the ability to sensitize cancer cells to cisplatin in two ways—by generating reactive oxygen species and by reducing the level of MRP1 protein, which is responsible for the removal of cisplatin–glutathione conjugates from cancer cells [101]. The increase in the level of reactive oxygen species caused by emodin enhances the cytotoxic effect of cisplatin, while the reduction in the level of MRP1 protein leads to an increase in the accumulation of cisplatin in the cell, thus increasing the effectiveness of its cytotoxic effect [101].

The group of quinones also includes another substance with anticancer activity—thymoquinone—a compound obtained from *Nigella sativa* L. [102]. This substance exhibits anticancer activity through a number of different mechanisms, among which we can distinguish the generation of reactive oxygen species, inhibition of the Nf-kB signaling pathway and activation of tumor-suppressor genes (p21 and PTEN) in cancer cells [102]. All these mechanisms potentiate the action of cisplatin, thus increasing the sensitivity of cancer cells to its action [102].

Another group of compounds with proven anticancer activity are phenols, among which genistein and luteolin deserve special attention [103,104].

Genistein is a compound obtained primarily from soybeans (lat. *Glycine Willd*) that show proapoptotic and antiproliferative effects against cancer cells [103]. It has been shown that the simultaneous treatment of cisplatin-resistant ovarian cancer cells with cisplatin and genistein leads to an increase in the effectiveness of cisplatin’s cytotoxic effect, by inactivating the Nf-kB signaling pathway by genistein. Inactivation of that pathway leads to the induction of apoptosis and inhibition of proliferation processes in cancer cells and, as a consequence, to the increase in their susceptibility to cisplatin [103].

Anticancer activity is also shown by another compound from the group of phenols—luteolin, the source of which are, for example, thyme (lat. *Thymus vulgaris* L.), rosemary (lat. *Rosmarinus officinalis*) and peppermint (lat. *Mentha xpiperita* L.). Luteolin is a substance that exhibits anticancer activity through a number of mechanisms, including inhibition of the cell cycle, induction of apoptosis or inhibition of angiogenesis [104]. It can be used to increase the sensitivity of ovarian cancer cells to cisplatin through its ability to inhibit the expression of the antiapoptotic protein Bcl-2, thus intensifying the process of apoptosis of cancer cells [104].

Another compound that has the ability to sensitize cells resistant to chemotherapeutic agents is saikosaponin-D—a substance obtained from the root of the *Bupleurum falcatum* L. [105]. It has been shown that saikosaponin-D increases the sensitivity of cisplatin-resistant ovarian cancer cells to the drug, by inducing mitochondrial fission and inhibiting the cell cycle of cancer cells in the G2/M phase. In addition, this substance causes an increase in the concentration of calcium ions in the cytosol of cancer cells, thus facilitating the loss of mitochondrial membrane potential, leading to the activation of the apoptosis process [105].

### 5.4. Combination Therapy with Cisplatin and Radiotherapy

The history of radiotherapy begins in 1985, when Wilhelm Roentgen discovered the existence of X-rays. Only a few months later, this type of radiation was first used for the treatment of cancer—first breast and skin cancers and then also other types of cancers [106,107]. The cytotoxic effect of radiation results from direct or indirect (caused by the reactive oxygen species generated as a result of water radiolysis) damage to the DNA of cancer cells, consequently leading to their apoptosis [106,107]. In addition, radiation used in sublethal doses may have a significant impact on the molecular processes in cancer cells [19]. It has been shown that pretreatment of both cisplatin-sensitive and cisplatin-resistant ovarian cancer cells with low-dose fraction radiation increases their sensitivity to the drug. Exposure of ovarian cancer cells to low-dose radiotherapy probably causes an increase in the expression of FOX3 protein—a transcription factor that plays an important role in the regulation of apoptosis, differentiation and metabolism of cells. In cisplatin-resistant cells, the level of FOX3 protein is significantly reduced; therefore, increasing its level has a positive effect on their sensitivity to cisplatin. For this reason, low-dose fraction radiotherapy may be an effective adjuvant therapy in the treatment of ovarian cancer [19].

### 5.5. Combination Therapy with Cisplatin and Hyperthermia

The increase in cancer cells’ sensitivity to cisplatin can also be achieved by the use of therapy combining cisplatin and hyperthermia. The exposure of cancer cells to elevated temperatures causes a number of molecular changes, which contribute to the inhibition of cisplatin-resistant mechanisms developed by these cells. For example, the exposure of cells to moderate hyperthermia leads to an increase in the fluidity of the cell membranes and to changes in their permeability, enabling the increased accumulation of cisplatin in these cells [108]. Moreover, when ovarian cancer cells are treated with elevated temperature, a significant increase in the intensity of the formation of cisplatin–DNA adducts is observed, which is probably caused by an increase in the accumulation of the drug in the cells or a decrease in the concentration of glutathione in them [108]. Furthermore, treating cancer cells with mild hyperthermia also leads to a decrease in the activity of DNA repair mechanisms (in particular, the nucleotide excision repair mechanism), which significantly reduces the intensity of removal of the cisplatin–DNA adducts formed as a result of cisplatin action [108]. As some results show the simultaneous treatment of cells with cisplatin and hyperthermia leads to a significant increase in the effectiveness of the cytotoxic effect of cisplatin on cancer cells, compared to the effectiveness obtained when cancer cells are treated only with cisplatin or only with hyperthermia [20].

Nowadays, a combination therapy using cisplatin and hyperthermia is used in the treatment of ovarian cancer in the form of hyperthermic intraperitoneal chemotherapy—HIPEC. HIPEC consists of the direct administration of a preheated to 40–43 °C, cisplatin solution, into the peritoneum of the abdominal cavity and pelvis. What is important is that the administration of the drug takes place immediately after cytoreductive surgery. The advantage of HIPEC therapy is 10–20 times higher drug concentration delivered directly to cancer cells, compared to intravenous chemotherapy [109]. The results of multiple studies show that patients who received HIPEC therapy showed a significant increase in overall survival time, as well as an increase in progression-free survival [109].

Despite the promising results of the so far conducted studies, the therapy with hyperthermia still needs further improvements, including the development of a method of homogenous tumor mass heating and protection of normal patient tissues from high temperatures [110].

### 5.6. Nanocarriers for Controlled Delivery of Cisplatin

A different approach to increase the effectiveness of cisplatin in the treatment of ovarian cancer is using nanocarriers for the controlled delivery of cisplatin to cancer cells. The drug delivery system using nanocarriers has a number of great advantages, including precise delivery of drugs to the cancer cells, the ability to adjust the size of the molecules, used in a drug delivery system, high loading capacity and stability of drug delivery molecules, as well as increased bioavailability of the drug delivered in this way [17,111,112].

One type of nanocarriers that can be used in the transport of cisplatin to ovarian cancer cells is lipid nanocarriers, which include nanostructured lipid carriers, liposomes and solid lipid nanoparticles. The main advantages of lipid nanocarriers as drug transporters are low toxicity, the ability to transport various types of drugs and the possibility to modify their surface [17,113,114,115]. An example of lipid nanocarriers that can be used to deliver cisplatin to ovarian cancer cells is PEGylated cisplatin-transporting liposomes, designed by Krieger et al. [116]. The results of the conducted studies indicate that the uptake of those liposomes was at the same level for both cisplatinum-sensitive and cisplatinum-resistant cells. The concentration of cisplatin in cells, to which the drug was administered via liposomes, was significantly higher than in cells to which the drug was administered directly, thus ensuring its higher cytotoxicity [116].

Another type of nanocarriers that can be used in the transport of cisplatin is polymeric nanocarriers. Those nanocarriers can be made of synthetic polymers (e.g., polylactide) or natural polymers (e.g., chitosan). The most important advantage of this type of nanocarriers is the possibility of releasing the drug from the carrier in a specific, controlled manner, e.g., continuously or depending on the occurrence of a given stimulus (e.g., low pH of the environment) [117,118]. Transport of cisplatin to cancer cells is possible, for example, with the use of polyamidoamine dendrimers (PAMAM), which deliver the drug to target cells with much greater efficiency than when the drug is administered in free form [119].

Nanocarriers can also be used to sensitize resistant cells to cisplatin, thus positively influencing its effectiveness. An example of such nanocarriers is magnetic iron oxide nanoparticles. It has been proven that these nanoparticles, by their ability to increase the level of reactive oxygen species in cancer cells (and thus lower the level of cellular glutathione), can increase the sensitivity of cancer cells to the action of cisplatin. This effect was also observed when using low doses of cisplatin, which may be helpful to reduce the side effects of the drug [120].

**Table 1:** Examples of combination therapies with cisplatin.

Components of Combination Therapy	Possible Applications	References
Cisplatin + paclitaxel	Ovarian cancer Cervical cancer Gastric cancer Non-small-cell lung cancer	[7,8,80,95]
Cisplatin + docetaxel	Ovarian cancer Head and neck cancer Non-small-cell lung cancer Breast cancer	[80,98,121,122]
Cisplatin + cardamonin	Ovarian cancer	[99]
Cisplatin + berberine	Ovarian cancer Breast cancer Gastric cancer	[100,123,124]
Cisplatin + emodin	Ovarian cancer Non-small-cell lung cancer Bladder cancer Gastric cancer Endometrial cancer	[101,125,126,127,128]
Cisplatin + thymoquinone	Ovarian cancer Lung cancer	[102,129]
Cisplatin + genistein	Ovarian cancer Cervical cancer Non-small-cell lung cancer	[103,130,131]
Cisplatin + luteolin	Ovarian cancer Colorectal cancer	[104,132]
Cisplatin + saikosaponin-D	Ovarian cancer Gastric cancer Cervical cancer Non-small-cell lung cancer	[105,133,134]
Cisplatin + radiotherapy	Ovarian cancer Head and neck cancer Cervical cancer	[19,120]
Cisplatin + hyperthermia	Ovarian cancer Bladder cancer Oral cancer	[108,135,136]

**Table 2:** Summary of approved and under-trial cisplatin analogs.

Platinum Complex	Possible Applications	Current Approval Status	References
Cisplatin	Ovarian cancer Testicular cancer Head and neck cancer Bladder cancer	Approved and used in cancer therapy since 1978	[6,85]
Carboplatin	Ovarian cancer Non-small-cell lung cancer Breast cancer Cervical cancer Testicular cancer	Approved and used in cancer therapy since 1989	[85,137,138]
Oxaliplatin	Colon cancer Gastric cancer Esophageal cancer Ovarian cancer Testicular cancer	Approved and used in cancer therapy since 1999 in Europe and 2002 in the USA	[85,91]
Nedaplatin	Non-small-cell lung cancer Esophageal cancer Head and neck cancer Ovarian cancer Urothelial cancer	In clinical trials. Approved and used in cancer therapy since 1995 in Japan	[85,139]
Heptaplatin	Gastric cancer Head and neck cancer	In clinical trials. Approved and used in cancer therapy since 2005 in Korea	[6,85]
Lobaplatin	Breast cancer Small-cell lung cancer Ovarian cancer Cervical cancer Gastric cancer	In clinical trials. Approved and used in cancer therapy since 2004 in China	[85,140,141]

## 6. Conclusions

Cisplatin is one of the most commonly used anticancer drugs, which is applied mostly in the treatment of ovarian, testicular, head and neck cancers, as well as bladder cancer. The action of this drug is based on the activation of apoptosis processes in cells, through several different mechanisms, which include damaging the genetic material of cancer cells (as a result of the formation of cisplatin–DNA adducts), induction of oxidative stress and damaging the mitochondria of those cells.

One of the most important disadvantages that are limiting the wider use of cisplatin in cancer therapy is the toxic side effects of its use, among which the most frequently observed are nephrotoxicity and ototoxicity, but also gastrointestinal toxicity or cardiotoxicity. Another very important problem related to the use of cisplatin is the development of resistance to the drug, which occurs in a large group of patients treated with it.

It has been proven that cancer cells have developed a number of mechanisms that protect them from the cytotoxic effects of cisplatin. The most important ones are changes in the activity of protein transporters, which cause a decrease in the cellular uptake of the drug and an increase in the export of it. In cisplatin-resistant cancer cells, an increase in the effectiveness of DNA damage repair mechanisms, as well as changes in the cytoskeleton and mitochondria, which contribute to the resistance, is also observed. Certainly, at this moment, we know only a part of the resistance mechanisms developed by cisplatin-resistant cells, so there is still a need to conduct further research on this topic, in order to treat patients suffering from this disease even more effectively.

Due to all these problems, methods of increasing the sensitivity of cancer cells to cisplatin and methods of improving the effectiveness of this drug are being intensively sought. High hopes are placed in combination therapies—mostly in therapies that combine cisplatin with other cytotoxic drugs (primarily paclitaxel and docetaxel), but also with the use of hyperthermia or radiotherapy, in particular low-dose fraction radiotherapy. In order to improve the effectiveness of cisplatin and, at the same time, reduce the side effects caused by the drug, a lot of attention is paid to the transport of it to cancer cells. One of the strategies that can potentially improve the drug transport is the use of nanocarriers—primarily lipid and polymer nanocarriers. For the same purpose, intensive research for cisplatin analogs is being carried out. Currently, two analogs are used in the treatment, carboplatin and oxaliplatin, and a couple more analogs are in clinical trials.

The presented review article is not intended to be just another publication repeating information about what cisplatin therapy is. It is intended to show the possibility of combining “classical” therapy based on a known compound with the improvements in treatment efficacy and safety that result from the latest scientific insights. Strategies, such as NGS techniques, molecular analysis of single cells, molecular positioning and the spatial “architecture” of the transcriptome of cancer tumors that is gradually being described (so-called single cell and spatial multiomics), are another set of new tools offered to researchers, thanks to these, and based on the obtained results, it will be possible to understand, replenish and offer new therapeutic solutions. Due to the amazing ability of cancer cells to avoid “obstacles” in the form of various types of therapeutic modulators introduced into these cells (including genotype–phenotype plasticity), each new piece of information allows to fill in the gaps, lay out a new picture or modify previously used therapeutic procedures. One can even assume that perfecting therapy, as we understand it today, is like filling in missing puzzles in the created picture.

From our point of view, the outline of this complex “picture” of anticancer therapy, including ovarian cancer therapy, is what we know about the mechanisms, effects and failures of platinum compound therapy, and what is supposed to improve this picture is the proposal of solutions, such as combination therapy or the use of unique carriers for cisplatin as a drug. Some of the treatment strategies presented in this article are already introduced into the treatment, while other ones are still in the clinical trial phase. Despite the great progress that has been made in the treatment of ovarian cancer, it is still necessary to continue further research, mainly on the effectiveness, but also on the safety of the described methods, and search for other, more advanced ways to treat this disease.

## Data Availability

Not applicable.
